# Comparison between ixazomib+cyclophosphamide+dexamethasone regimen and ixazomib+dexamethasone regimen for elderly and frail patients having newly diagnosed multiple myeloma

**DOI:** 10.1002/cam4.5422

**Published:** 2022-11-15

**Authors:** Shutan Li, Duanzhong Zhang, Lihua Yang, Chunlan Huang, Ting Niu, Yuping Gong

**Affiliations:** ^1^ Department of Hematology, West China Hospital Sichuan University Chengdu Sichuan Province People's Republic of China; ^2^ Department of Hematology, Dazhou Central Hospital Dazhou city Sichuan Province People's Republic of China; ^3^ Department of Hematology Affiliated Hospital of Southwest Medical University Luzhou Sichuan People's Republic of China

**Keywords:** elderly, frail, multiple myeloma, oral ixazomib

## Abstract

**Aims:**

The purpose of this prospective, randomized study was to investigate the effectiveness and safety of the ixazomib+cyclophosphamide+dexamethasone (ICd) and ixazomib+dexamethasone (Id) regimens in newly diagnosed multiple myeloma (NDMM) who were elderly and frail and to compare the two regimens.

**Methods:**

Patients were randomly grouped into ICd and Id group. The primary end point was ORR, and patients who received at least two cycles were analyzed. The median follow‐up was 13.5 months. After nine induction cycles, patients were instructed to take single ixazomib for maintenance.

**Results:**

The overall response rate in the ICd and Id groups was 78.9% and 70.6%, respectively, whereas the very good partial remission or better rate was 47.4% and 23.5%, respectively. For the ICd and Id groups, the response rate after 4 cycles was 76.5% and 57.1%, and the median duration to response was 2 and 4 months, respectively. Adverse events (AEs) included gastrointestinal intolerance, rash, fatigue, and thrombocytopenia, with severe AEs occurring in 21.1% and 23.5% patients in the ICd and Id groups, respectively, and the AEs were manageable. Both the QLQ‐C30 and QLQ‐MY20 scales indicated that ICd and Id regimens could help maintain and improve the quality of life(QoL).

**Conclusions:**

The ICd and Id regimens might be effective and well‐tolerated in elderly and frail patients with NDMM. In addition, a favorable outcome was observed that ICd might tend to cause faster and higher remission than Id regimen without increasing the risk of AEs. The long‐term effectiveness and safety of the two regimens need further investigation.

## Graphical abstract

ICd regimen followed by ixazomib maintenance therapy is effective, safe, convenient, and well‐tolerated in elderly and transplant‐ineligible NDMM patients.

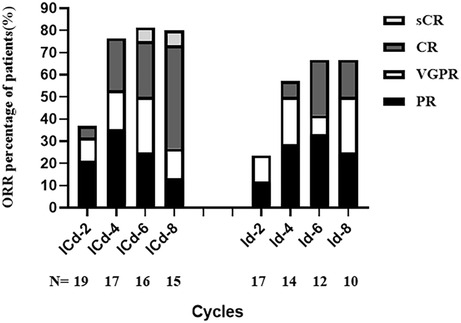

## 1 INTRODUCTION

Multiple myeloma (MM) generally occurs in senior individuals. At diagnosis, the median age of MM patients is 69 years, with 33% of them having an age over 75 years.^1^ Many individuals having newly diagnosed MM (NDMM) are frail, exhibit severe comorbidities, and are commonly not eligible for intensive treatments including autologous stem cell transplantation (ASCT). Age is an important factor to assess frailty for MM, while the geriatric assessment (GA) is a more sensitive predictor of frailty. The International Myeloma Working Group (IMWG) develops GA for elderly MM patients,^2^, ^3^, ^4^ which includes age, Lawton and Brody's instrumental ADL (IADL) scale, Katz and Akpom's basic activities of daily living (ADL) scale, and the Charlson Comorbidity Index (CCI). Based on GA, Palumbo A provides an easy and quick online score system to calculate the frailty score. Patients with MM are classified into fit (score = 0), intermediate‐fit (score = 1), and frail (score ≥ 2).^2^ Compared with the fit and intermediate‐fit patients, the frail has a worse prognosis.^2^, ^3^

There are no standard regimens for the elderly and frail patients with NDMM.^1^ Hence, new, effective, safe, and suitable regimens for older frail MM patients are needed.^5^ With the development of immune modulators (IMIDs; lenalidomide, pomalidomide) and proteasome inhibitors (PIs; bortezomib, ixazomib and carfilzomib), the management of MM has considerably improved. Triplet regimens comprising PIs, IMIDs and dexamethasone have become the common treatment for NDMM patients,^6^ for example, the bortezomib +lenalidomide+dexamethasone (RVD) regimen has achieved very good efficacy.^7^ As the standard frontline therapy for MM, RVD is generally effective and safe, but it may be not well tolerated and convenient in some elderly and frail. For example, IMIDs may be not preferred for some patients because of severe renal impairment, potential toxicity,^8^ venous thromboembolism,^9^ probability of second primary malignancies,^10^ and so on. Combinations without IMIDs, such as carfilzomib or bortezomib with melphalan‐prednisone (KMP or VMP) or cyclophosphamide‐dexamethasone (KCD or VCD), as well as a combination of ixazomib and MP, are efficacious in NDMM treatment.^11^, ^12^, ^13^, ^14^, ^15^, ^16^, ^17^, ^18^, ^19^ However, these different combinations may have limited feasibility in varying aspects for some elderly and frail patients because of the potential risk of peripheral neuropathy (PN) and renal or cardiac injury, the requirement of regular intravenous or subcutaneous administration,^11^, ^14^, ^18^ or the limitation of medical insurance in China. In addition, the related guidelines for the elderly and frail patients are also limited after progression. Elderly and frail NDMM patients are prone to adverse events (AEs), and may be less tolerant to the same regimens than the fit, or intermediate‐fit patients, which may cause early discontinuations of treatments or reductions in drug dosage.^16^, ^17^, ^18^, ^19^ Meanwhile, MM in the elderly and frail patients is heterogeneous, and different individuals may have different tolerance to the different regimens. Clinical trials for these patients are underrepresented,^1^ and there are no unified regimens at present for induction and progression.

As the first PI administered orally, ixazomib has been approved for MM patients who have undergone prior treatments with fewer AEs.^15^, ^20^ Combinations of weekly ixazomib regimens have indicated good therapeutic effects in MM patients.^21^, ^22^ Meanwhile, maintenance therapy is important in the current MM therapeutic approaches for better clinical outcomes.^23^, ^24^, ^25^, ^26^ Efficacious regimens with limited toxicity, easy administration for prolonged periods, and the ability to maintain the quality of life (QoL) are needed. Oral ixazomib has the advantages of safety and convenient administration, but it is more expensive than bortezomib in China, and only one of the IMIDs and PIs can be included in medicare reimbursement. Hence, the initial regimen of an oral ixazomib‐based combination comprising ixazomib, low‐dose dexamethasone (ICd) and cyclophosphamide (ICd) or low‐dose dexamethasone and ixazomib (Id) is probably more tolerable and convenient for the elderly and frail patients with NDMM. Herein, we recruited the frail patients and investigated the efficacy and adverse effects of ICd and Id in the induction period administered along with ixazomib as single‐agent maintenance therapy in senior and fragile patients with NDMM. The treatment for progression need another investigation in the future, which is not the intention in this preliminary study.

## 2 METHODOLOGY

### 2.1 Trial design

Our study was a prospective and randomized study. The ICd group was the experimental group, and the Id was the control group. Patients were randomly assigned to receive the ICd or Id regimen according to a random number table. Neither the investigator nor the patients could speculate on which group the next patients would be assigned to, and the person who determined the random number table would not participate in the inclusion of the patients. The grouping information was kept strictly.

**FIGURE 1:**
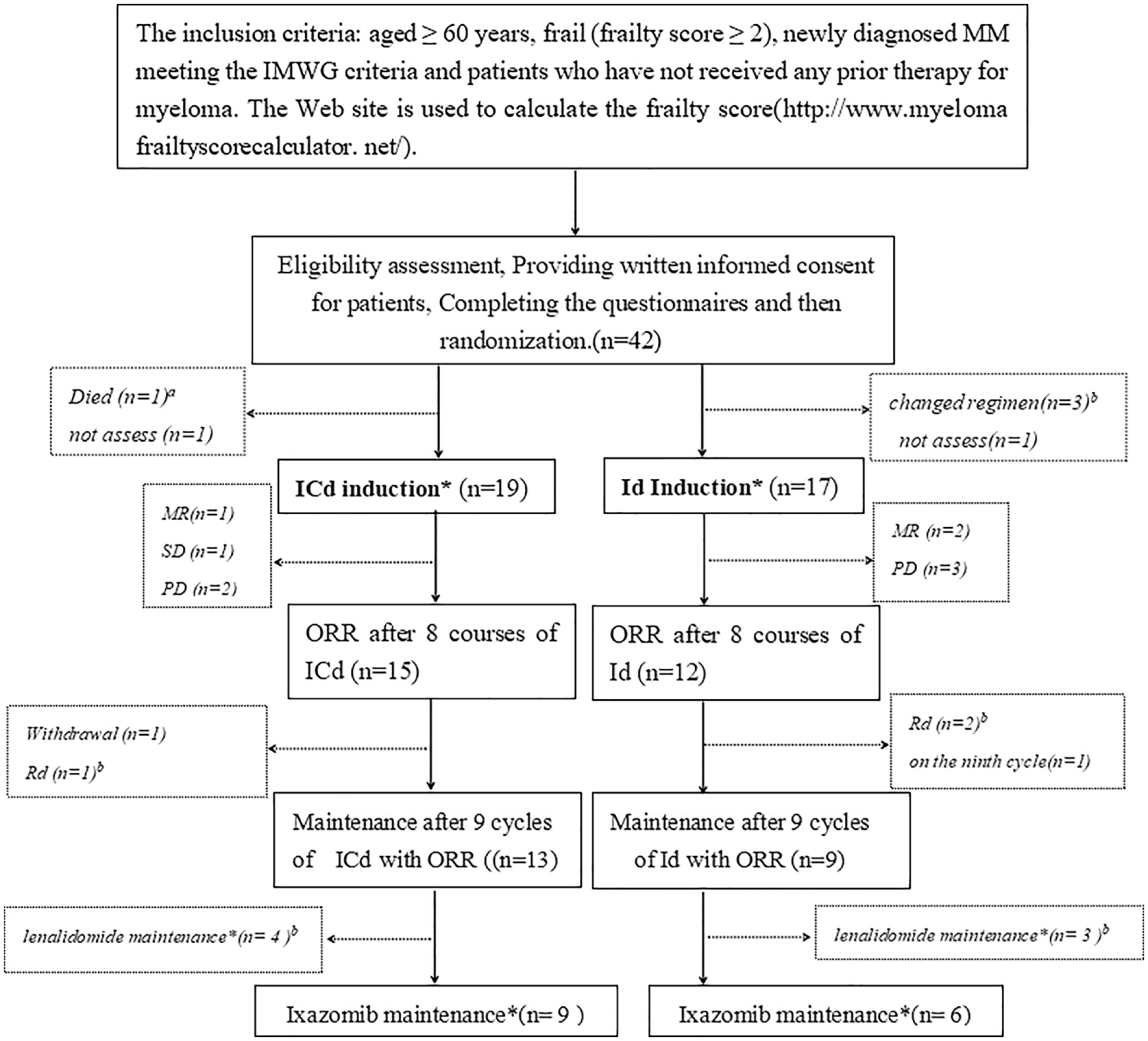
Trial flow diagram. Id, ixazomib+dexamethasone; ICd, ixazomib+cyclophosphamide+dexamethasone. ORR, Partial remission or better. Withdrawal standard*, poor response, disease progression, death, and severe toxicity or requirement of patient. *a*,one died during the first course of chemotherapy because of heart and kidney failure caused by multiple myeloma. *b*, switched to cheaper regimens for economic considerations.

Patients who received at least two cycles were analyzed in this initial study. During the treatment, patients would withdraw from this study once there was poor response, progression of disease, death or drug intolerance. Patients who experienced progression would be assessed further, and subsequently be administered the appropriate regimens according to the possible mechanisms of resistance,^27^ potential adverse effects and the related contraindications. The detailed design is shown in Figure 1.

### 2.2 Patients

This prospective, randomized trial was conducted at three centers: West China Hospital of Sichuan University, Affiliated Hospital of Southwest Medical University, and Dazhou Central Hospital. The inclusion criteria were as follow: ≥60 years of age, frail (frailty score ≥ 2), newly diagnosed MM meeting the IMWG^27^ criteria and patients who had not received any prior therapy for myeloma. We used the website (http://www.myelomafrailtyscore calculator.net/) to calculate the frailty score, based on which all the patients in this study were frail (score ≥ 2).

As ixazomib was more expensive than bortezomib in China, many patients who met the eligibility criteria refused to enroll in the clinical trial because of financial and health insurance concerns. Overall, 42 patients were included in this study from July 2020 to June 2022. Among them, 36 patients (19 in the ixazomib+cyclophosphamide+Dexamethasone [ICd] group and 17 in the ixazomib+dexamethasone [Id] group) had received at least two cycles of therapy, and they were further assessed. The last day of the observation period was June 30, 2022, and the study is ongoing.

The study was approved by the Ethics Committee of West China Hospital of Sichuan University and review boards at all institutions that participated in this study. The research was performed in compliance with the the Guidelines for Good Clinical Practice, the Guidelines of the International Conference on Harmonization, and the Declaration of Helsinki. Informed consent was obtained from all the participants before the initiation of the study.

### 2.3 Treatments

The patients were randomly grouped to receive either the ICd or Id regimen during the induction stage. Because the patients were old and frail, dexamethasone and cyclophosphamide were administered with reduced doses. The dosage of ixazomib was adjusted to 3 mg for elderly patients with worse comorbidities and more adverse effects to the drug to select an appropriate dose. Detailed regimens were as follows: patients in the ICd group received 4 or 3 mg ixazomib (on the 1st, 8th and 15th days), 10 mg dexamethasone (on the 1st, 2nd, 8th, 9th, 15th, 16th, 22nd, and 23rd days), and 200 mg/m^2^ cyclophosphamide (on the 1st, 8th, 15th, and 22th days). In the Id group, the doses of ixazomib and dexamethasone were the same as those in the ICd group; each cycle consisted of 28 days. After 9 cycles of induction, patients with partial response (PR) or better were administered single‐agent ixazomib as maintenance therapy on the 1st, 8th and 15th days per cycle, 28 days per cycle, until the disease progressed, death, or intolerable toxicity.

### 2.4 M protein estimation

The levels of monoclonal protein were important in the diagnosis and assessment of efficiency, so serum protein electrophoresis (SPE), immunofixed electrophoresis (IFE), serum free light chain quantification, urine light chain quantification and 24 h urine M protein quantification were used for M protein estimation. M protein type of IgG, IgA, IgM, IgD, IgE, λ or κ was identified by immunofixation electrophoresis (IFE). The M protein levels of the patients were examined every two cycles, and the change of M protein level was used to judge the therapeutic effect.

According to IMWG,^27^ M protein criteria for NDMM was monoclonal protein≥10 g/L for IgG, ≥5 g/L for IgD, IgE, IgM and IgA, in the serum; Or urinary level of monoclonal protein ≥200 mg/24 h; Or free light chain (FLC) concentration ≥ 100 mg/L and abnormal FLC ratio of Kappa/lambda in the serum. The main standard of M protein for efficacy evaluation was complete remission (CR: M protein was negative in the serum and urine for immunofixation); very good partial remission (VGPR: positive serum and urinary M proteins were detected by immunofixation, and electrophoresis detected a reduction of ≥90% in serum M protein and a reduction in urinary M protein ≤100 mg/24 h); partial remission (PR: serum M protein decreased by ≥50%, 24 h urinary level of M protein decreased by ≥90% or reduced to ≤200 mg/24 h); minimal remission (MR: serum M protein reduced by ≥25% but ≤49%, and reduction of urinary M protein 50%–89% in 24 h).

### 2.5 Assessment of effectiveness and safety

Diagnosis, disease stage, therapeutic efficacy, and the progression of disease were all evaluated based on the IMWG criteria.^27^ The required baseline assessments performed before randomization included physical examination; evaluation of the Eastern Cooperative Oncology Group performance status and medical history; Durie‐Salmon, International Staging System (ISS), and revised‐ISS stage; and hematological, biochemical, and bone marrow sample tests. The participants received regimens unless the following events occurred: poor response, progression of disease, death, drug intolerance or toxicity, or withdrawal by consent. During the treatment, the regimen was changed if no MR was observed after the fourth cycle or no PR was observed after the sixth cycle or in case of disease progression or intolerance. The patients who received at least two courses were included in the safety and efficacy analyses. The primary outcomes were the combined overall rate of response (ORR, also being referred to as partial response or better [≥PR]) and the rate of very good partial response or better (≥VGPR) after ICd or Id administration. Other data such as the rate of complete response, duration from the first drug administration to the onset of a response, decreased degree of M‐protein response, progression‐free survival, safety, and associated AEs were recorded. We graded the AEs based on the National Cancer Institute Common Terminology Criteria for Adverse Events (CTCAE‐Version 5.0).

In addition, the participants completed the questionnaires of EORTC QLQ‐C30 QoL and QLQ‐MY20 questionnaires before and after therapy, which helped in assessing the change in quality of life (QoL). The EORTC QLQ‐C30 contains 30 items. These items are included the scale of Global Health QoL, 3 scales evaluating the symptoms (pain, fatigue, and nausea or vomiting), 5 scales assessing functioning of patients (physical functions, emotional functions, cognitive functions, role functioning and social functioning), and 6 items determining insomnia, dyspnea, appetite loss, constipation, diarrhea, and financial difficulties of patients. Higher scores of the functional and QoL scales represented better QoL; whereas for the symptom scale and 6 independent items, a higher score denotes poor patients' QoL. The EORTC QLQ‐MY20 is a questionnaire specifically adjusted for patients with MM, and it can supplement the QLQ‐C30 questionnaire.^28^ It consists of 20 items concerning four HRQoL domains specific to myeloma: 1. “Disease Symptoms” covering pain in the chest, back, arm or shoulder, hip, or bone and pain intensifying with activity; 2: “Side Effects of Treatment,” covering tingling in the hands or feet, restlessness or agitation, sleepiness, ill feeling, dry mouth, thirst, acid indigestion or heartburn, burning or sore eyes, hair loss, and upset by hair loss; 3: “Future Perspectives,” covering thinking about illness and worrying about future health and death; and 4: “Body Image.” A higher score for each item indicates poor functioning.^28^, ^29^

### 2.6 Statistical analyses

Categorical variables were described as counts and percentages, and continuous variables were described as medians and ranges. Comparisons of categorical variables were performed by Fisher's exact test, and continuous variables were compared by the Mann–Whitney's U test. The Wilcoxon signed rank test was used for defining significant changes in QoL before versus after therapy. PFS was calculated by the Kaplan–Meier test for univariate analyses. The value of α was 0.05, two‐tailed, and *p* < 0.05 was considered to indicate statistical significance. Based on the formulas [N = (μ_α_ + μ_β_)^2^ (1 + 1/*k*) *P* (1−*P*)/(*P*_*e*_−*P*_*c*_)^2^, *P* = (*P*_*e+*_ *kP*_*C*_)/(1 + *k*)], we calculated the power value of the ORR between the two groups. SPSS v.21.0 and GraphPad Prism 9 were utilized to carry out the statistical analyses.

## 3 RESULTS

### 3.1 Basic characteristics of patients

According to the inclusion criteria, we enrolled 42 patients. The patients who received at least two‐cycle therapy were assessed. Following patients were excluded: one patient died during the first cycle of chemotherapy because of heart and kidney failure caused by multiple myeloma, three patients switched to cheaper regimens for economic reasons after one cycle, and two patients had not finished the first or second cycle, respectively. As patients who received at least two cycles were analyzed in this study, these six patients were excluded. Therefore, a total of 36 patients from three centers in China were assessed in this study. Among them, 19 patients were included in the ICd group and 17 in the Id group. The median follow‐up duration was 13.5 months (range: 2–24 months). The median age was 75 years (range: 60–87 years); 52.8% of patients were ≥ 75 years old and 41.7% of the patients were men. Furthermore, 75.0% and 61.1% of patients had anemia and renal impairment in various degrees, respectively. The ISS III stage was identified in 55.6% of the patients. The detailed characteristics of the patients at baseline are presented in Table 1.

**TABLE 1:** Baseline clinical characteristics of the patients

Characteristic	ICd (*n* = 19)	Id (*n* = 17)	*p*	Overall (*n* = 36)
Age				
X ± SD	73.7 ± 8.2	76.2 ± 6.9	0.320	74.9 ± 7.6
Median (years, range)	74(60–83)	77 (63–87)	0.446	75(60–87)
Years < 65 (*n*, %)	5 (26.3)	2 (11.8)	0.956^a^	7 (19.4)
65 ≤ years < 75 (*n*, %)	5 (26.3)	4(29.4)		10 (27.8)
Years ≥ 75 (*n*,%)	9 (47.4)	11(58.8)	0.789	19(52.8)
Male sex‐(*n*, %)	9 (47.4)	6(35.3)	0.516	15 (41.7)
ECOG score‐*n*(%)				
1	5 (26.3)	3 (17.6)	0.695	8 (22.2)
2	9 (47.4)	10 (58.8)	0.525	19 (52.8)
3	4 (21.1)	4 (23.5)	1.000	8(22.2)
4	1 (5.3)	—	—	1 (2.8)
≥2	14(73.7)	14(82.4)	0.695	28(77.8)
Myeloma disease type‐*n* (%)				
IgG	13 (68.4)	8 (47.1)	0.311	21(58.3)
IgA	4 (21.1)	4 (23.5)	1.000	8 (22.2)
Kappa	—	2 (11.8)	—	2 (5.6)
Lamda	2 (10.5)	3 (17.6)	0.650	5 (13.9)
DS stage‐*n* (%)				
I	1 (5.3)	—	—	1 (2.8)
II	5 (26.3)	6 (35.3)	0.721	11(30.6)
III	13 (68.4)	11 (64.7)	1.000	24 (66.7)
ISS stage‐*n* (%)				
I	1 (5.3)	2 (11.8)	0.593	3 (8.3)
II	8 (42.1)	5 (29.4)	0.502	13(36.1)
III	10 (52.6)	10 (58.8)	0.749	20 (55.6)
R‐ISS stage‐*n* (%)				
I	—	1 (6.7)	—	1 (2.8)
II	5 (26.3)	5 (29.4)	1.000	10 (27.8)
III	9 (47.4)	6(35.3)	0.516	15(41.7)
Data not available	5 (26.3)	5 (29.4)	1.000	10(27.8)
Cytogenetic features‐*n* (%)				
Standard‐risk abnormalities^b^	7 (36.8)	5 (29.4)	0.732	12 (33.3)
High‐risk abnormalities^c^	7 (36.8)	7 (41.2)	1.000	14 (38.9)
Data not available	5 (26.3)	5 (29.4)	1.000	10 (27.8)
Complications before treatment				
eCCR (ml/min)‐*n*(%)				
50–80	7 (36.8)	3 (17.6)	0.274	10 (27.8)
31–50	2 (10.5)	5 (29.4)	0.219	7 (19.4)
<30	2 (10.5)	3 (17.6)	0.650	5 (13.9)
Cardiovascular/pulmonary Comorbidity‐*n*(%)	9 (47.3)	10 (58.8)	0.525	19 (52.8)
Hemoglobing/L, ‐*n* (%))				
≥90	6 (31.6)	2 (11.8)	0.236	8 (22.2)
60–90	6 (31.6)	7 (41.2)	0.730	13(36.1)
<60	3 (15.8)	3 (17.6)	1.000	6 (16.7)
≥3 Lytic bone disease‐*n* (%)	9 (47.4)	8 (47.1)	**0.000**	17 (47.2)
Median time (range) ‐mo	14 (2–24)	13(2–18)	0.332	13.5 (2–24)

*Note*: eCCr = (140−Age) × Mass (kg) × [0.85 if female]/72 × [Serum Creatinine (mg/dl)].

Abbreviations: DS, Durie‐Salmon; eCCr, estimated creatinine clearance rate; ECOG, Eastern Cooperative Oncology Group; Ig, immunoglobulin; ISS, International Staging System; R‐ISS, Revised International Staging System.

^a^ <75 years.

^b^ Includes Trisomy, *t* (11, 14), *t* (6;14), and others.

^c^ Includes *t* (4;14), *t* (14;16), *t* (14;20), deletion 17p, gain 1q, or p53 mutation.

## 4 RESPONSE TO THERAPY AND EFFICIENCY

### 4.1 ORR, VGPR and better response rate

All the patients were evaluated and administered at least two courses of treatment. In addition, the ixazomib dose was adjusted to 3 mg for 4 and 5 patients from the ICd and Id groups, respectively. Meanwhile, patients would withdraw from this trial after progression. Progression in both groups during the induction was considered to be disease progression (PD) when we analyzed the response in the induction phase. In fact, in the induction phase, two patients in the ICd group progressed after 5 and 8 cycles, respectively, and three patients in the Id group progressed after 2,3 and 7 cycles, respectively. Regardless of their disease gauge after switching to other regimens, we considered them as PD (Table 2), which ensures the rational evaluation of response in induction therapy of both groups.

**TABLE 2:** Assessment of confirmed response to ICd or Id induction therapy

Response –*n* (%)	ICd (*n* = 19)	Id (*n* = 17)	*p*	Total (*n* = 36)
sCR	2 (10.5)	—	0.408^a^	2 (5.6)
CR	3 (15.8)	2 (11.8)		5 (13.9)
VGPR	4 (21.1)	2 (11.8)	0.662	6(16.7)
PR	6(31.6)	8(47.1)	0.495	14 (38.9)
MR	1 (5.3)	2(11.8)	0.593	3(8.3)
SD	1(5.3)	—	1.000	1(2.8)
PD	2(10.5)	3 (17.6)	0.650	5(13.9)
≥VGPR	9(47.4)	4 (23.5)	0.177	13(36.1)
ORR^b^ (≥PR)	15 (78.9)	12(70.6)	0.706	27(75.0)

*Note*: ORR, PR, VGPR, CR, sCR, MR, SD, PD^27^; n, the total number of patients.

^a^ CR + sCR.

^b^ The power of ORR (ICd vs Id) was 0.875, which was calculated by using the formulas, *N* = (*μ*_α_ + *μ*_β_)^2^ (1 + 1/*k*) *P* (1−*P*)/(*P*_*e*_−*p*_*c*_)^2^, *P* = (*P*_*e*_ + *kP*_*C*_) / (1 + *k*); [*P*_*e*_ = 0.789, *P*_*C*_ = 0.706, *N* = 36, the alpha was 0.05, two‐ tailed, *μ*_α_ = 1.96].

Overall, 75.0% of patients achieved the ORR in the entire trial, and 15 (78.9%) and 12 (70.6%) patients from the ICd and Id groups achieved a confirmed ORR (≥PR), respectively, and the value of the power between the two groups was 0.875 (Table 2); 47.4% and 23.5% patients of the ICd and Id groups displayed ≥VGPR, respectively. However, no *p* value was <0.05. The detailed responses for the patients are shown in Table 2.

### 4.2 ORR with disease stage and age

The ORR of the ICd group in ISS stages III, II, and I was 70.0%, 87.5%, and 100.0%, respectively, whereas the corresponding ORR of the Id group was 60.0%, 80.0%, and 100.0%. Meanwhile, patients in ISS stage III (65.0%) had lower ORR than those in stage II (84.6%) and I (100.0%) in both groups. Comparing the ORR of different stages in the two groups, it was not statistically significant (Table S1).

The mean age of the ICd group is 73.7 years, while that of the Id group was 76.2 years, and the median age of ICd group was 74 years, while that of the Id group was 77 years. There were 10 (52.6%) patients in the ICd group who were aged <75 years and only 6 (35.3%) patients in Id the group who were aged <75 years. Overall, patients in ICd seemed to be younger than those in the Id group, and the percentage of patients aged ≥75 years was higher in the Id group than in the ICd group. Age is quite an important prognostic factor of MM, so we analyzed whether the difference in efficacy of ICd and Id might be attributed to the younger population in the ICd group. It seemed that the ORR was higher in the ICd group (77.8%) than in the Id group (60%) when patients were ≥75 years old, and slightly lower in the ICd group (80%) than in the Id group (85.7%) when they were <75 years old. However, there was no significant difference (*p* = 0.628, *p* = 1.000, respectively; Table S1). In addition, there was no significant difference in age between the ICd group and the Id group (*p* = 0.446, Table 1), and there was no difference between the two groups when the age was ≥75 (*p* = 0.789, Table 1) or <75 years (*p* = 0.956, Table 1). There were more women in both groups, especially in the Id group, while the ORR in different gender had no difference not only in ICd group but in the Id group (*p* = 1.000, Table S1).

### 4.3 Response of patients at the different ends of treatment cycles

**FIGURE 2:**
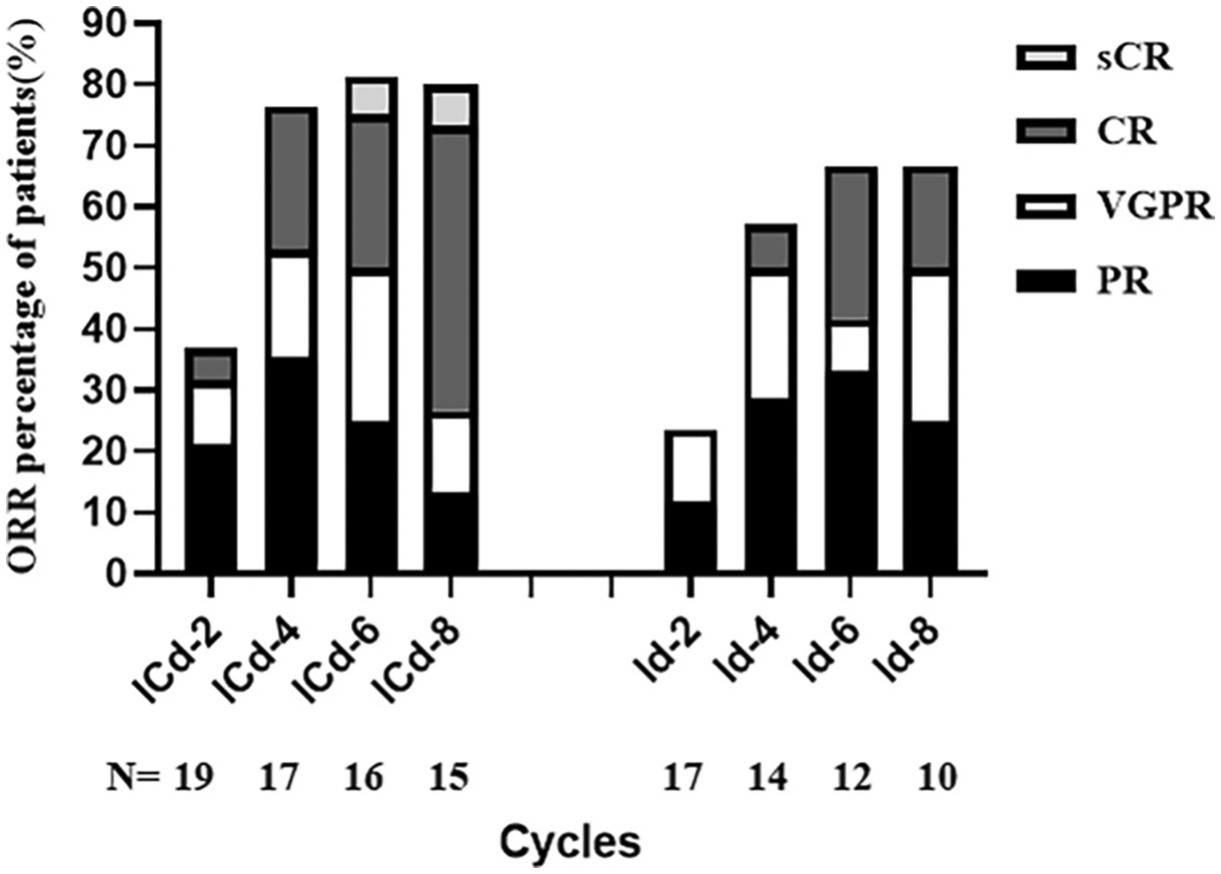
Relationship between ORR and treatment cycles in ICd group and Id group. ORR: response of PR or better, objective response rate, including PR(partial response) VGPR (very good partial response), CR(complete response) and sCR(stringent complete response); Id: ixazomib+dexamethasone. ICd: ixazomib+cyclophosphamide+dexamethasone. *p* > 0.05.

The median durations for the first ≥PR were 2 (range: 2–5 months) and 4 (range: 2–6 months) months in the ICd and Id groups, respectively. An improved response was observed as the patients received more treatment cycles (Figure 2). The ICd and Id groups exhibited response rates of 76.5% and 57.1%, respectively, at the end of the fourth cycle. As prespecified criteria for efficacy were not met, one patient in the ICd group was changed to VRd, and 2 patients in the Id group were subjected to the ICd or Bd regimen (Figure 1). Thirteen patients in the ICd group and 9 patients in the Id group received single‐agent maintenance therapy. However, only 9 patients in the ICd group and 6 in the Id group were finally treated with ixazomib for maintenance, whereas 4 patients in the ICd and 3 patients in the Id group received lenalidomide for maintenance therapy for financial reasons (Figure 1).

### 4.4 Changes in M‐protein response

**FIGURE 3:**
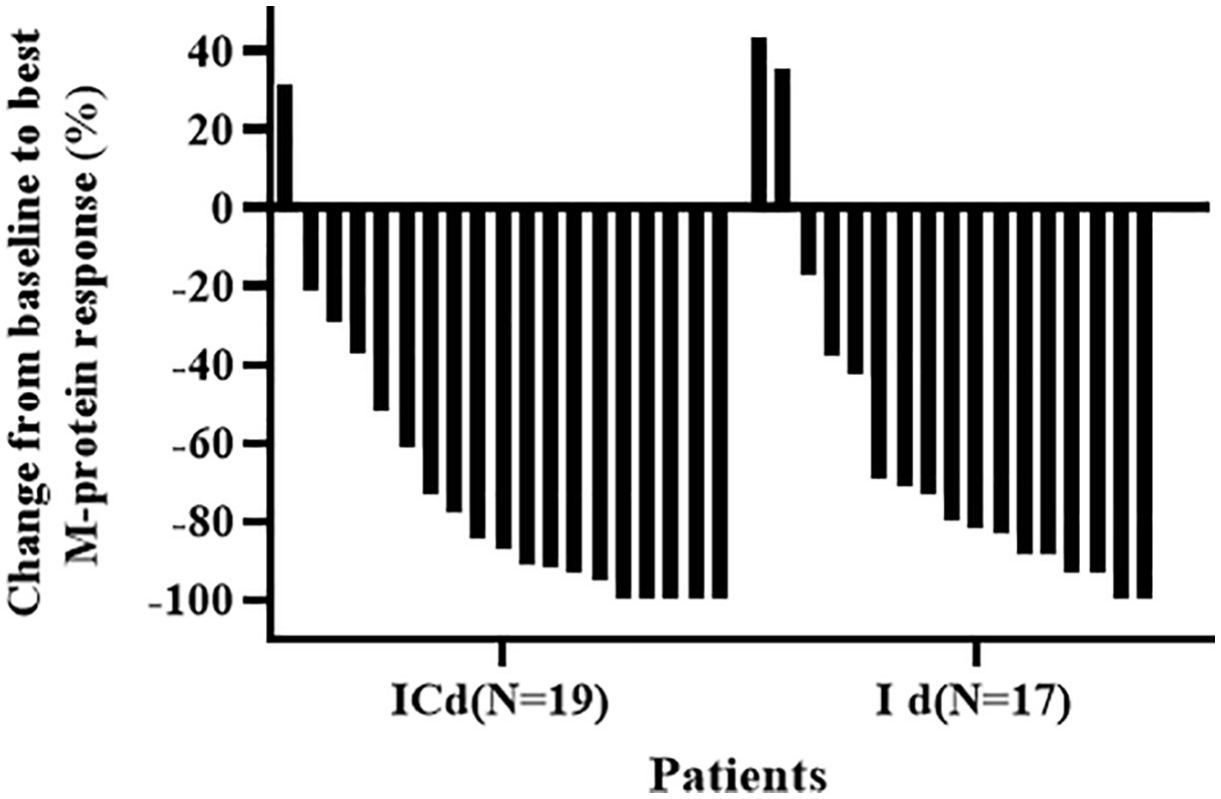
Changes of M‐protein from basline to best response during the treatment of ICd or Id regimen. M‐protein: monoclonal immunoglobulin.

Changes from the baseline to the best M‐protein response (%) in this study were as follows: M‐protein response decreased by ≥ 90% and ≥ 50% in 36.1% and 75.0% in all the patients; M‐protein response decreased by ≥90%, 50%–90%, and ≥ 50% in 47.4%, 31.6% and 78.9% patients in the ICd group, respectively, and in 23.5%, 47.1%, and 70.6% patients in the Id group, respectively (Figure 3).

### 4.5 Improvement in comorbidity

In the ICd group, 11 patients had kidney injury at diagnosis (Table 1), whereas 5 patients displayed CR and 1 patient displayed PR after 1–5 cycles; 4 patients were stabilized, and the condition of 1 patient deteriorated. Additionally, 15 patients had anemia at diagnosis, of which 5 recovered, 5 improved after 1–7 cycles, 4 with mild anemia were stable, and the condition of 1 patient deteriorated. In the Id group, in terms of kidney injury, 4 and 3 patients achieved CR and PR, respectively, after 2–6 cycles; 3 patients were stable, and the condition of 1 patient deteriorated (*n* = 11). In terms of anemia, 5 patients recovered after 2–4 cycles, 3 displayed improvement, 2 were stable, and 2 exhibited deteriorated conditions (*n* = 12). In conclusion, the recovery rates of renal function were 54.5% and 63.6% in the ICd and Id groups, respectively. The anemia improvement rates in both groups were 66.7%.

### 4.6 Prognosis of the disease

**FIGURE 4:**
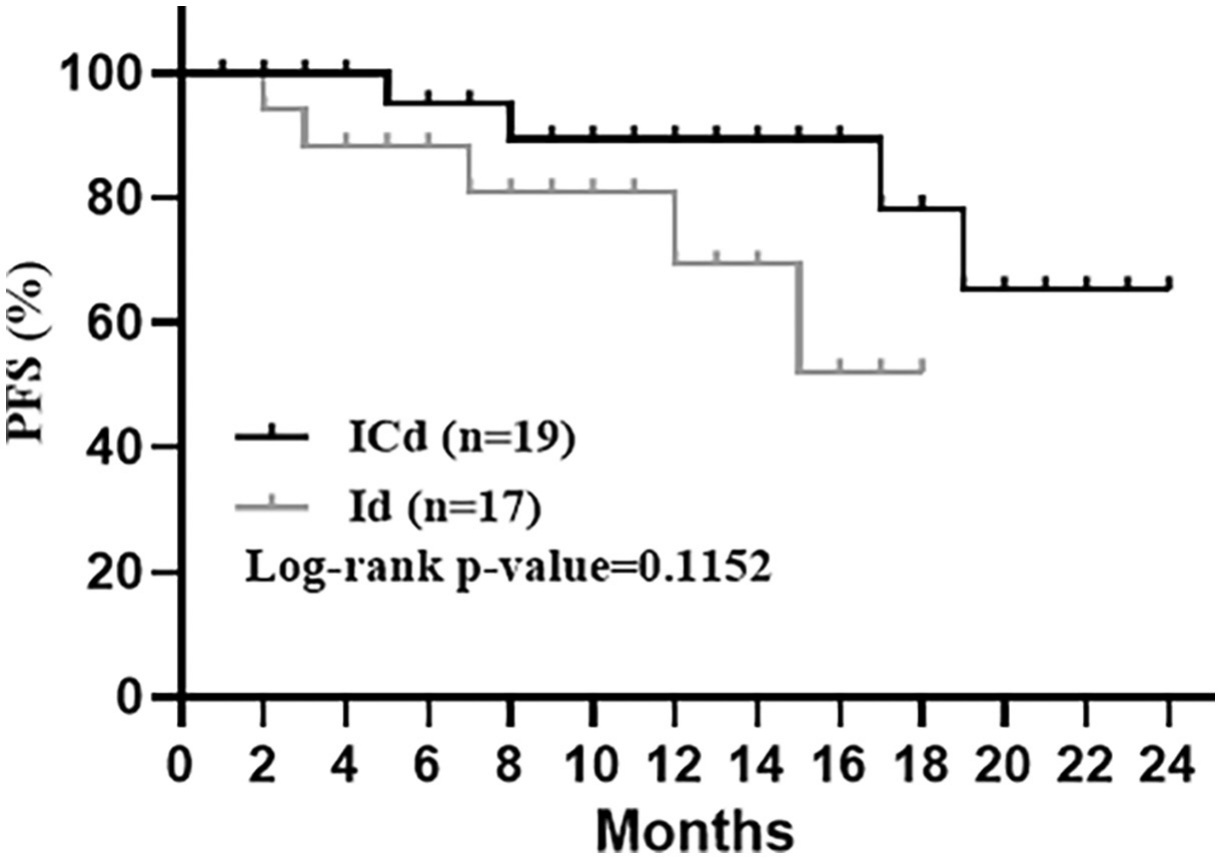
Progression‐free survival (PFS) by ICd and Id treatment. PFS rates at the endpoint were 78.9% and 70.6% in ICd and Id, respectively. Kaplan–Meier analysis, *p* > 0.05.

Because the follow‐up time was short and no patient died during the study, no overall survival analyses could be performed. Four patients in the ICd group exhibited disease progression after 5,8,17 or 19 cycles, respectively, whereas five patients in the Id group exhibited progression after 2, 3, 7,12 or 15 cycles, respectively, (Figure 4).

### 4.7 Safety and adverse events

The most frequently observed toxicity events were grades 1 and 2 gastrointestinal disorders, incidences of rashes, thrombocytopenia and fatigue, followed by loss of appetite, abdominal distension, vomiting, and banded blister disease, which occurred mainly in the first three cycles. The incidence rates of grades 3 and 4 AEs in the ICd and Id groups were 21.1% and 23.5%, respectively. In the ICd group, one patient developed grade 3 thrombocytopenia, and three patients developed severe digestive AEs (two patients exhibited diarrhea, another exhibited vomiting accompanied by abdominal distension). In the Id group, one patient had abdominal distension, one exhibited vomiting and two had diarrhea (Table 3).

**TABLE 3:** Adverse events in the ICd and Id groups

*n* (%)	ICd (*n* = 19)		Id (*n* = 17)		Total (*n* = 33)	
AEs	Grde 3 or 4^a^	Total	Grade 3 or 4^a^	Total	Grade 3 or 4	Total
Thrombocytopenia	1	4 (21.1)	0	2 (11.8)	1	6(16.7)
Leucopenia	0	1 (5.3)	0	2 (11.8)	0	3(8.3)
Loss of appetite	0	3 (15.8)	0	3 (17.6)	0	6(16.7)
Vomiting	1	3 (15.8)	1	3 (17.6)	2	6(16.7)
Diarrhea	2	8(42.1)	2	7(41.2)	4	15(41.7)
Abdominal distension	0	2 (10.5)	1	4 (23.5)	1	6(16.7)
Infection	0	1 (5.3)	0	2(11.8)	0	3(8.3)
Banded blister disease	0	3 (15.8)	0	2 (11.8)	0	5(13.9)
Peripheral neuropathy	0	2 (10.5)	0	1 (5.9)	0	3(8.3)
Rash	0	6 (31.6)	0	4 (23.5)	0	10(27.8)
Fatigue	0	4(21.1)	0	4 (23.5)	0	8(22.2)

*Note*: Adverse event criteria (CTCAE ‐ Version 5.0) to assess the severity of adverse events of chemotherapies. ICd vs Id.

^a^ *p* = 1.000; *p* > 0.05.

### 4.8 QoL evaluated based on the scores of the EORTC QLQ‐C30 and QLQ‐MY20

The EORTC QLQ‐C30 showed that the items of physical functioning, fatigue and pain had been improved significantly in both groups after chemotherapy. In addition, the values for global health status and dyspnea in the ICd group were improved. However, financial difficulties increased in the ICd group after chemotherapy (Table S2).

Based on the analysis of the QLQ‐MY20 scale, we observed significant improvement in disease symptoms and body image in the ICd group after treatment. In the Id group, the improvement in disease symptoms was significant after chemotherapy (Table S3).

## 5 DISCUSSION

Studies have reported that the treatment response in elderly patients is poorer than that in young patients,^2^, ^30^ partially because the elderly are more fragile.^23^, ^31^ GA is a highly sensitive predictor of frailty. The GA recommended by the IMWG integrates the factors such age, comorbidities, cognitive and physical conditions, which can predict mortality and the risk of toxicity in elderly frail myeloma patients, and propose treatment decisions. Moreover, frail patients display poor compliance and unwillingness to visit hospitals. The outcomes of MM have been improved substantially in recent years.^31^ Triplet regimens based on IMs and PIs are more efficacious than doublet regimens,^7^, ^32^, ^33^, ^34^ but PIs and IMIDs cannot be included in medical insurance together in China. Hence, effective agents are needed^23^ in these patients. Ixazomib has the advantages of good efficacy, few adverse effects, convenient administration and good compliance. Cyclophosphamide provides an important choice for MM in some cases. Therefore, we investigated the efficacy and safety of ICd and Id regimens in elderly and fragile patients with NDMM.

### 5.1 The effectiveness of the ICd and Id regimens

There are few studies on ICd in NDMM, and no studies have compared ICd with Id, especially in the elderly and frail patients with NDMM. Our results indicated an overall ORR of 75.0%, indicating that oral ICd and Id could provide good tolerance and therapeutic effects in the elderly and frail patients with NDMM. In particular, comparisons between Id and ICd were conducted. The ORR of ICd and Id regimens was 78.9% and 70.6%, respectively, and the power value of the ORR between the two groups is was 0.875. Although the number of patients included is currently small, the power is >0.8. After four cycles, the rate of ≥PR in the ICd and Id groups was 76.5% and 57.1%, respectively, whereas the corresponding ≥VGPR rates were 47.4% and 23.5%, respectively. The ICd regimen seemed to result in faster remission and higher ORR than the Id regimen, and additional cycles could improve the depth of response (Figures 2 and 3 and Table 2), but the number of patients was small, and we will enroll more patients to further confirm it. In addition, we observed the younger population in the ICd group than in the Id group. Patients in ICd had a higher ORR and the ORR was higher in the ICd group than in the Id group when the age was ≥75 years. However, we could still not conclude that the ICd regimen was significantly superior to the Id regimen in different age groups significantly (Table S1). The effect of the ICd regimen on the ORR in different age groups needs further investigation with larger sample size, and further assessment will be continued.

The ICd regimen exhibited promising efficacy in NDMM.^21^, ^35^ In the study by Dimopoulos, 67 patients were evaluated and the median age was 73 (61–87) years. Most of the patients had cardiopulmonary diseases and renal insufficiency, but the ECOG score was ≤1 in 81% of patients. In the study by Kumar, 48 patients were evaluated; the median age was 64.5 (41–88) years, and the ECOG score was 0–2. After the median treatment duration of 19 cycles by Dimopoulos and a median follow‐up of 25.6 months by Kumar, the ORR was 76% and 77%, respectively. Compared with the studies by Dimopoulos^35^ and Kumar,^21^ only 36 elderly and frail patients receiving at least two cycles were assessed in this study and the number of patients in our study was smaller with a shorter follow‐up. However, all the patients recruited in our study were older and frail and the frailty score was calculated. The ECOG score was ≥2 in 80% of patients. Thus, our patients might be older and more fragile and had worse conditions at diagnosis. In addition, our patients received ICd therapy with reduced doses of both cyclophosphamide and dexamethasone, but a similar ORR was acquired. In brief, ICd might be an effective regimen in the elderly and frail patients with NDMM, although the response rates might be lower than other three‐drug combinations: One trial demonstrated that the ORR was 92.1%,^36^ and in another study, it was 82.1% after administration of IRd^15^; 95.2% ORR was observed in patients treated with VCd^37^; The ORR of KCd was 95%.^14^ We suspected that the different drug combinations or inclusion criterias might affect the response; on the other hand, we will continue the study to acquire more significant results.

### 5.2 The adverse effects (AEs) of the ICd and Id regimens

Ixazomib was safe and the common toxicities included leucopenia, thrombocytopenia, nausea, diarrhea, vomiting, and erythema‐multiforme,^21^, ^35^ most of which were ≤2 grades. Our patients mainly presented with digestive tract discomfort, skin rash and thrombocytopenia, which were well‐tolerated and manageable. AEs occurred mainly in the first three cycles but were relieved after dose modifications and supportive care measures. The median treatment duration was 14 months, which indicated the long‐term tolerance to the therapy and the multiple cycles of therapy did not seem to result in noticeable cumulative toxicity. In addition, no more AEs were observed in the ICd group than in the Id group.

Studies by Dimopoulos^35^ and Kumar^21^ had reported that the incidences of grade ≥3 AEs were 73% and 71%, respectively, which were higher than that of 22.2% (ICd 21.4%; Id 23.5%) in our study. Meaghan reported that the incidences of grade ≥3 AEs with RD, CRD, and VCD were 61%, 74%, and 41%,^8^ respectively, and the toxicities ≥grade 3 with VRD and VCD were 76% and 79%, respectively.^12^ In the TOURMALINE‐MM2 trial, 88% and 81% of patients experienced grade ≥3 AEs^15^ in the IRd and Rd groups, respectively. Thus, the incidences of grade ≥3 AEs in our study seemed to be lower. This might be related to the fact that we reduced the doses of cyclophosphamide and dexamethasone. In addition, we deduced that it might be related to the bias of the patient visits, and the different observation time. Further, the QoL scores remained stable and improved after therapy in both groups (Table S2,S3). Therefore, our preliminary results showed that the tolerability to these regimens in elderly fragile patients with NDMM.

### 5.3 Limitations of this study

This was the first study to use geriatric assessment to investigate ICd and Id in elderly frail patients with NDMM. All the enrolled patients in this study were frail (score ≥2), and the results provided evidence for treatment of the elderly and frail patients with NDMM, but there were some limitations in this study. First, the sample size was small and no robust conclusions could be drawn. Second, the follow‐up was short and not sufficient to observe the long‐term outcomes and the prognosis. Third, the number of patients receiving ixazomib for maintenance therapy was small and relevant conclusions could not be drawn. Fourth, generally, gender does not affect the ORR and prognosis of MM, but our data was biased towards female. Whether gender had an effect on the results requires further study. In addition, we could not conclude the effects of cytogenetics, stages and age on the prognosis significantly. Finally, as we mainly assessed patients who received at least two cycles of treatment, there might be some bias. A larger number of patients will be enrolled to further study, which will give a more comprehensive real‐world analysis including the clinical and economic benefits. In brief, we made descriptive statistics for the study due to the small number of patients, and made no more convincing conclusions about the relevance of ICd and Id therapy. Although some results were not statistically significant, this initial study presented a real‐world analysis of the combinations, and the phenomenon between the two groups should not be ignored, which would provide potential clinical information.

## 6 CONCLUSIONS

There was a trend that the oral ICd or Id regimen exhibited good effectiveness and tolerability during the initial treatment of fragile patients with NDMM. Although no robust conclusions and comparison could be drawn with ICd and Id regimens regarding the small number of patients, a favorable outcome was observed in this trial. Moreove, the convenience and tolerability might make ixazomib effective in the long‐term treatment. To conclude, the promising clinical activity warrants further study. As an ongoing prospective study, we are expanding the samples to further study the effectiveness and safety of ICd followed by ixazomib as maintenance in elderly and fragile myeloma, as well as whether it is superior to the Id regimen.

## AUTHOR CONTRIBUTIONS

**Shutan Li:** Conceptualization (lead); data curation (lead); formal analysis (lead); investigation (lead); methodology (lead); project administration (supporting); resources (lead); validation (lead); visualization (supporting); writing – original draft (lead); writing – review and editing (lead). **Duanzhong Zhang:** Data curation (equal); project administration (equal); supervision (equal); writing – original draft (supporting). **Lihua Yang:** Conceptualization (equal); data curation (equal); supervision (equal); writing – original draft (equal). **Chunlan Huang:** Data curation (equal); supervision (equal); writing – review and editing (equal). **Ting Niu:** Data curation (equal); supervision (equal); writing – original draft (equal). **Yuping Gong:** Conceptualization (equal); data curation (lead); formal analysis (lead); funding acquisition (lead); investigation (equal); methodology (lead); project administration (lead); resources (equal); software (equal); supervision (lead); validation (equal); visualization (equal); writing – original draft (equal); writing – review and editing (equal).

All authors recruited patients; YP G designed this study; YP G and ST L collected and analyzed the data;ST L drafted the manuscript and all authors critically reviewed the manuscript and approved for publication.

## FUNDING INFORMATION

The research is funded by the Science and Technology Department of Sichuan Province, China (No. 2019YFS0026).

## CONFLICT OF INTEREST

We declare there are no potential conflicts of interest.

## THE CLINICAL TRIAL REGISTRATION

This research is registered at the website of Chinese clinical trial registry (ChiECRCT20200449). http://www.chictr.org.cn/showproj.aspx?proj=64839.

## ETHICS APPROVAL STATEMENT

The study was approved by the Ethics Committee of West China Hospital of Sichuan University and review boards at all participating institutions.

## PATIENT CONSENT STATEMENT

Before being recruited for the study, we obtained the written informed consent from all the participants.

## Supporting information


Table S1.
Table S2.
Table S3.

## Data Availability

This trial was initiated by the investigators. As the study is still ongoing, the data can not be provided at this time. The data availability will be available from the corresponding author.
