# Supplementary material for: Comparison between ixazomib+cyclophosphamide+dexamethasone regimen and ixazomib+dexamethasone regimen for elderly and frail patients having newly diagnosed multiple myeloma
Source: Cancer Med. 2022 Nov 15;12(6):6523–35. doi: 10.1002/cam4.5422 (PMC10067049; doi:10.1002/cam4.5422)
Supplement: Supplementary file 1 — Table S1. Table S2. Table S3. [file CAM4-12-6523-s001.doc]

**Table S1 Overall rate of response and disease stage**

| Stage/ORR | ICd (n = 19) | Id (n = 17) | P | Total (n = 36) |
| --- | --- | --- | --- | --- |
| DS stage — n/total (%) |  |  |  |  |
| I | 1/1 (100.0) | - | - | 1/1 (100.0) |
| II | 4/5 (80.0) | 5/6(83.3) | 0.706 | 9/11(81.8) |
| III | 10/13 (76.9) | 7/11(63.6) | 0.659 | 17/24 (70.8) |
| ISS stage —n/total (%) |  |  |  |  |
| I | 1/1 (100.0) | 2/2 (100.0) | - | 3/3 (100.0) |
| II | 7/8 (87.5) | 4/5 (80.0) | 1.000 | 11/13 (84.6) |
| III | 7/10 (70.0) | 6/10(60.0) | 1.000 | 13/20 (65.0) |
| R-ISS stage — n/total (%) |  |  |  |  |
| I | - | 1/1 (100.0) | - | 1/1 (100.0) |
| II | 4/5 (80.0) | 4/5 (80.0) | - | 8/10 (80.0) |
| III | 7/9 (77.8) | 4/6 (66.7) | 1.000 | 11/15 (73.3) |
| Overall Response Rate by FISH |  |  |  |  |
| High risk | 4/7 (57.1) | 4/7 (57.1) | 1.000 | 7/12 (57.1) |
| Standard risk | 5/7 (71.4) | 3/5 (60.0) | 1.000 | 8/12 (66.7) |
| Gender— n/total (%) |  |  |  |  |
| male | 7/9（77.8） | 4/6（66.7） | 1.000 | 11/15 (73.3） |
| female | 8/10（80.0） | 8/11（72.7） | 1.000 | 16/21(76.2) |
| Age (years)— n/total (%) |  |  |  |  |
| ＜75(n,%) | 8/10 (80.0) | 6/7 (85.7) | 1.000 | 14/17 (82.4) |
| ≥75(n,%) | 7/9 (77.8) | 6/10 (60.0) | 0.628 | 13/19 (68.4) |

DS, Durie-Salmon; ISS, International Staging System; R-ISS, revised International Staging System. p＞0.05.

**Table S2 Mean (MS) and standard deviation (SD) for EORTC QLQ-C30**

**scores in MM patients before versus after treatment**

|  | ICd–before *vs* after  chemotherapy  （n=19,MS±SD） | | p |  | Id–before *vs* after  chemotherapy  （n=17,MS±SD） | | p |  |
| --- | --- | --- | --- | --- | --- | --- | --- | --- |
|  | Before | After |  |  | Before | After |  |  |
| Global health status | 51.9±27.4 | 53.3±23.9 | **0.018**^*^ |  | 51.3±22.8 | 56.6±27.8 | 0.142 |  |
| Physical functioning | 40.7±22.3 | 50.2±21.2 | **0.003**^*^ |  | 52.2±20.4 | 54.5±21.2 | **0.014**^*^ |  |
| Role functioning | 42.1±27.8 | 52.6±24.9 | 0.094 |  | 44.7±27.1 | 57.9±23.6 | 0.093 |  |
| Emotional functioning | 48.7±19.1 | 53.5±16.7 | 0.169 |  | 46.1±18.9 | 51.3±20.4 | 0.131 |  |
| Cognitive functioning | 64.0±21.7 | 65.8±23.9 | 0.617 |  | 61.4±19.3 | 66.7±22.2 | 0.177 |  |
| Social functioning | 52.6±21.7 | 61.4±20.0 | 0.203 |  | 62.7±20.9 | 68.7±21.1 | 0.458 |  |
| Fatigue | 52.6±31.7 | 25.1±28.9 | **0.003**^*^ |  | 33.1±27.7 | 27.4±23.3 | **0.003**^*^ |  |
| Nausea/  vomiting | 9.5±23.0 | 14.3±23.1 | 0.197 |  | 10.8±24.2 | 14.7±24.2 | 0.285 |  |
| Pain | 28.9±33.6 | 14.0±25.6 | **0.006**^*^ |  | 36.1±26.0 | 24.1±25.1 | **0.002**^*^ |  |
| Dyspnea | 19.3±27.9 | 15.8±29.1 | **0.025**^*^ |  | 20.1±35.6 | 14.7±29.4 | 0.317 |  |
| Insomnia | 26.3±34.8 | 31.6±38.0 | 0.480 |  | 26.5±35.9 | 17.7±30.3 | 0.257 |  |
| Appetite loss | 21.1±30.3 | 28.9±30.3 | 0.317 |  | 17.6±30.3 | 26.5±31.2 | 0.366 |  |
| Constipation | 17.5±25.7 | 19.3±25.6 | 0.655 |  | 23.5±25.7 | 27.5±24.2 | 0.589 |  |
| Diarrhea | 13.2±28.0 | 17.5±25.8 | 0.096 |  | 8.8±26.4 | 17.6±30.3 | 0.180 |  |
| Financial difficulties | 29.8±31.2 | 36.8±37.6 | **0.022**^*^ |  | 27.9±27.9 | 41.2±41.4 | 0.700 |  |

Wilcoxon signed rank test, p^*^ ＜ 0.05.

**Table S3 Mean (MS) and standard deviation (SD) for QLQ-MY20 scores in MM patients before versus after treatment**

|  | ICd–before *vs* after  chemotherapy  （n=19,MS±SD | | p | Id–before *vs* fter  chemotherapy  （n=17,MS±SD） | | p |
| --- | --- | --- | --- | --- | --- | --- |
|  | Before | After |  | Before | After |  |
| Future concerns | 2.2±0.7 | 1.9±0.7 | 0.068 | 2.1±0.5 | 1.9±0.5 | 0.736 |
| Body image | 2.2±0.9 | 1.6±0.6 | **0.005**^*^ | 2.2±0.7 | 1.8±0.7 | 0.058 |
| Disease symptoms | 2.3±0.8 | 1.8±0.7 | **0.001**^*^ | 2.3±0.7 | 2.1±0.7 | **0.042**^*^ |
| AEs of treatment | 2.0±0.6 | 2.3±0.8 | 0.123 | 2.2±0.6 | 2.1±0.6 | 0.112 |

AEs, adverse effects; Wilcoxon signed rank test, p^*^ ＜ 0.05.
